# Supplementary material for: Change in Liver Fibrosis Associates with Progress of Diabetic Nephropathy in Patients with Nonalcoholic Fatty Liver Disease
Source: Nutrients. 2023 Jul 22;15(14):3248. doi: 10.3390/nu15143248 (PMC10386534; doi:10.3390/nu15143248)
Supplement: Supplementary file 1 [file nutrients-15-03248-s001.zip › nutrients-2444606-supplementary.pdf]

Table S1. Baseline characteristics of the patients according to FIB-4 index.

	FIB-4 Index		
	Low Risk Group n = 37	Indeterminate Risk Group n = 95	High Risk Group n = 57
Age, years	58 (42–74)	67 (61–73)	68 (61–75)
Female, n (%)	32 (32)	26 (35.6)	3 (18.8)
Body weight, kg	69.2 (54.8–83.6)	64.5 (50.4–78.6)	66.6 (52–81.2)
BMI, kg/m^2	25.9 (21.4–30.4)	25.5 (21.3–29.8)	25.8 (22.3–29.3)
Systolic blood pressure, mmHg	134 (118–150)	136 (115–157)	130 (107–154)
Diastolic blood pressure, mmHg	80 (67–93)	74 (58–90)	71 (60–83)
Hypertension, n (%)	58 (58)	46 (63)	10 (62.5)
Dyslipidemia, n (%)	28 (28)	29 (39.7)	3 (18.8)
HbA1c, %	7.6 (6.9–8.3) *	7.2 (6.7–7.7)	6.8 (6.2–7.4)
Creatinine, mg/dL	0.9 (0.59–1.21)	0.92 (0.59–1.25) *	0.86 (0.5–1.23)
eGFR, mL/min/1.73 m^2	65 (37.3–92.8)	56 (34.5–77.5) *	66 (41.5–90.5)
U-Alb/U-Cre, mg/g	83.5 (61.5–187.3)	97.5 (64–39.2)	123.1 (78–350.4
U-Pro/U-Cre, mg/g	1.4 (1.2–3.7)	1.3 (1.1–2.5)	0.75 (0.7–2.2)
DN stage, stage 2/stage 3 (%)	68/32, (68/32)	49/24 (67.1/32.9)	10/6 (62.5/37.5)
Estimated salt intake, g/day	10.6 (7.1–14.2)	10.6 (8–13.2)	11.7 (9.7–13.7)
AST, U/L	19 (12–28) *	21 (13–29)	27.5 (4–52)
ALT, U/L	24 (8–44)	19 (11–31)	22 (18–61)
Platelet count, ×10^3/μL	24.9 (18.9–30.8) *	19.7 (14.9–24.5)	13.8 (10.1–17.4)
FIB-4 index	0.99 (0.86–1.19) **†	1.68 (1.42–2.14) *	2.76 (2.26–3.66)
SGLT2 inhibitor, n (%)	0 (0)	0 (0)	0 (0)
GLP-1 agonist, n (%)	8 (8)	6 (8.2)	2 (12.5)
DPP-4 inhibitor, n (%)	48 (48)	39 (53.4)	9 (56.3)
Pioglitazone, n (%)	7 (7)	5 (6.8)	0 (0)
Insulin injection, n (%)	48 (48)	27 (37)	6 (37.5)

FIB-4 index <1.3 was a low risk, 1.3–2.66 was an indeterminate risk, and ≥2.67 was a high risk. The U-Alb/U-Cre ratio was measured in patients with DN stage 2. The U-Pro/U-Cre ratio was measured in patients with DN stage 3. The Dunn–Bonferroni test was used for the comparison of continuous variables between the groups. Fisher's exact test with the Bonferroni correction was used for the com-parison of categorical variables between the groups. **Abbreviations:** BMI—body mass index; HbA1c—hemoglobin A1c; eGFR—estimated glomerular filtration rate; U-Alb/U-Cre—urine albumin to urine creatinine ratio; U-Pro/U-Cre—urine protein to urine creatine ratio; DN—diabetic nephropathy; AST—aspartate aminotransferase; ALT—alanine aminotransferase; FIB-4—fibrosis 4; SGLT2—sodium glucose cotransporter 2; GLP-1—glucagon-like peptide-1; DPP4—dipeptidyl peptidase 4. * $p < 0.05$ and ** $p < 0.001$, compared with the high risk group; † $p < 0.05$ and †† $p < 0.001$, compared with the indeterminate risk group.

Table S2. Regression analysis of baseline characteristics for changes in FIB-4 index.

	Explanatory Variable	Partial Regression Coefficient	*t* Value	*p* Value	95% C.I
Univariate					
	Gender	0.033	0.444	0.675	−0.137–0.216
	Age	−0.037	−0.487	0.627	−0.012–0.007
	BMI	−0.053	−0.703	0.483	−0.027–0.013
	Systolic blood pressure	−0.172	−2.312	0.022	−0.013–-0.001
	Diastolic blood pressure	−0.091	−1.215	0.226	−0.013–0.003
	Hypertension	0.063	0.84	0.402	−0.098–0.243
	Dyslipidemia	−0.052	−0.688	0.492	−0.242–0.117
	HbA1c	−0.112	−1.482	0.140	−0.131–0.019
	eGFR	−0.078	−1.033	0.303	−0.007–0.002
	DN category	−0.154	−2.071	0.040	−0.361–0.009
	Estimated salt intake	−0.113	−1.44	0.152	−0.064–0.01
Multivariate					
	Systolic blood pressure	−0.155	−2.077	0.0392	−0.012–0.001
	DN category	−0.135	−1.809	0.0721	−0.337–0.015

A simple regression model analysis and multiple regression model analysis. **Abbreviations:** BMI—body mass index; HbA1c—hemoglobin A1c; eGFR—estimated glomerular filtration rate; DN—diabetic nephropathy; AST—aspartate aminotransferase; ALT—alanine aminotransferase; FIB-4—fibrosis 4.

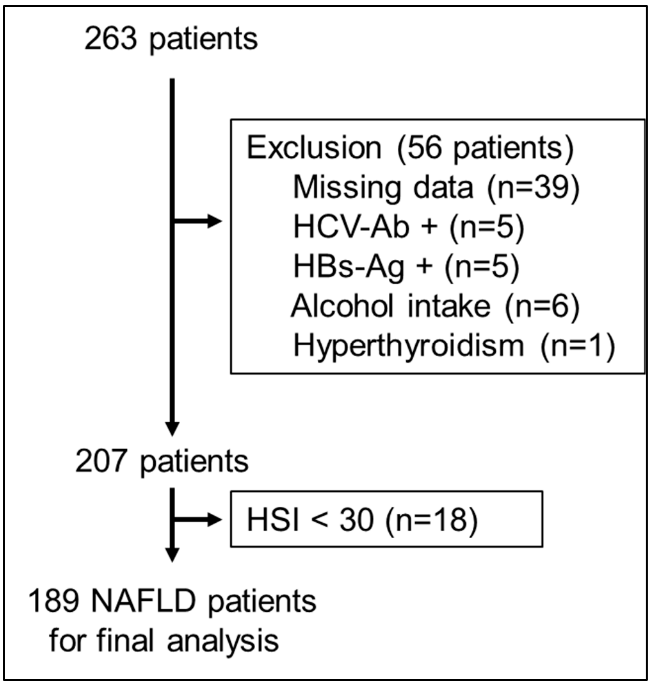

Figure S1. Patient flow of the study. A total of 56 patients were excluded from analysis due to missing data and possible etiology which affects liver function. Information about alcohol intake was based on interviews by the physician and nurse in charge. **Abbreviations:** HCV-Ab—hepatitis C virus antibody, mass index; HBs-Ag—hepatitis B surface antigen; HSI—hepatic steatosis index; NAFLD—nonalcoholic fatty liver disease.
